# Adaptive Ferrofluidic Robotic System with Passive Component Activation Capabilities

**DOI:** 10.34133/cbsystems.0300

**Published:** 2025-06-24

**Authors:** Qinkai Chen, Haozhe Feng, Xinjian Fan, Hui Xie, Lining Sun, Zhan Yang

**Affiliations:** ^1^ School of Future Science and Engineering, Soochow University, Suzhou 215222, China.; ^2^ School of Mechanical and Electrical Engineering, Soochow University, Suzhou 215131, China.; ^3^ State Key Laboratory of Robotics and Systems, Harbin Institute of Technology, Harbin 150080, China.

## Abstract

Soft robots demonstrate remarkable potential in medical applications owing to their minimally invasive nature, exceptional controllability, and shape-adaptive capabilities. However, existing control systems primarily rely on a single permanent magnet or electromagnetic coil for actuation, resulting in limited robotic motion capabilities, weak electromagnetic field gradient forces, and bulky magnetic drive systems. These constraints substantially hinder the robot’s flexibility and functional expandability. To address these constraints, this study proposes a highly integrated hybrid electromagnetic coil permanent magnet actuation system. This innovative design enables actuation force amplification and synergistic regulation of locomotion, deformation, and orientation. Experimental validation confirms the broad operational capacity of the miniature ferrofluidic robot (MFR), including controllable motion-deformation coupling within multiscale luminal structures and active directional control in biomimetic gastric models. Leveraging the MFR’s robust deformation and locomotion abilities, the empowerment mechanism for passive structures significantly enhanced compatibility with mechanical systems. Based on this mechanism, we achieved the transportation of larger-mass simulated drug particles by empowering passive delivery systems. To further validate the functionality of MFR, we developed an MFR-based capsule that achieves precise temporal and spatial control of drug release through experiments involving magnetothermal effect-accelerated release of simulated drugs and selective occlusion in simulated blood vessels. These advancements markedly enhanced the application potential of microrobots in complex and confined clinical environments.

## Introduction

Magnetically controlled miniature robots offer a paradigm shift in biomedical interventions, surpassing the limitations of traditional mechanical approaches and targeted therapies [1–4]. These microscopic devices, manipulated with precision by magnetic fields, exhibit remarkable potential for revolutionizing treatments and minimally invasive surgeries [5–8]. For instance, the 5-degree-of-freedom electromagnetic driving system for intraocular microsurgery [9], the rotating multibarreled capsule robot for multiple biopsies and on-demand drug delivery [10], and the programmable soft microgel robot with encoded magnetization [11] are capable of performing complex tasks in biomedical environments, such as drug delivery and surgical procedures, markedly reducing the risks and invasiveness associated with conventional methods [12–15]. These robots can access previously unreachable internal regions, performing treatments, diagnostics, and targeted drug delivery with minimal collateral damage, thereby enhancing the safety and efficiency of medical interventions [8]. Furthermore, their inherent magnetic propulsion system enables superior 3-dimensional (3D) navigation and reduces invasiveness, optimizing therapeutic accuracy and patient comfort [16,17]. Recent advancements in micro/nanorobots have yielded substantial progress, particularly in developing miniature ferrofluidic robots [18,19]. Unlike conventional approaches employing permanent magnetic materials or elastomers, ferrofluidic robots exhibit exceptional deformability and adaptability to complex biological environments [20]. Innovations in materials, including integrating magnetic fluids into hydrogels, have enhanced the biocompatibility and maneuverability of miniature ferrofluidic robots (MFRs) [21–23]. Notably, researchers have developed novel actuators for clearing blockages, investigated fluid dynamics within intestinal capsules [24], designed highly dexterous robots for intravascular navigation [25], and achieved complex motion control through light-responsive materials [26].

Despite these achievements, MFRs encounter challenges related to driving systems and functional integration. Traditional permanent magnet-based drive devices can generate strong gradient magnetic fields but are unable to produce flexible rotating magnetic fields [27]. On the other hand, drive devices based on electromagnetic coils, such as Helmholtz coils and distributed electromagnetic coils, can generate flexible rotating magnetic fields but are incapable of producing large gradient magnetic fields, making it difficult to control millimeter-scale magnetic micro-robots precisely [19]. Additionally, commonly used systems such as Helmholtz coils and distributed electromagnetic coils tend to have large sizes, and their workspaces are limited [28]. This restricts the application of magnetic control devices in confined spaces, such as in medical robotic arms where a more compact design is necessary [29,30]. Moreover, in recent years, mobile electromagnetic actuation devices have notably emerged. For instance, Cai et al. [31] developed a reconfigurable electromagnetic actuation system by integrating 3 electromagnetic coils on independent robotic arms. Additionally, Yang et al. [32] proposed a parallel-mobile-coil system for magnetic actuation. These systems enable distributed electromagnetic coil arrays to move within 3D space and drive robotic motion. However, relying solely on electromagnetic coils for magnetic field generation introduces several challenges. First, generating sufficient gradient fields and magnetic moments to actuate robots necessitates large-scale installations and high energy consumption, which compromises the compact functionality inherent to mobile magnetic control systems. Second, the limited magnetic field strength produced by electromagnetic coils, combined with the integration constraints of mobile platforms, exacerbates field instability. Third, such field instability and insufficient field intensity inevitably prolong feedback latency during robot locomotion and directional adjustments. These limitations collectively hinder the practical application of mobile electromagnetic systems in scenarios requiring high-precision control and rapid dynamic responses.

**Fig. 1.**
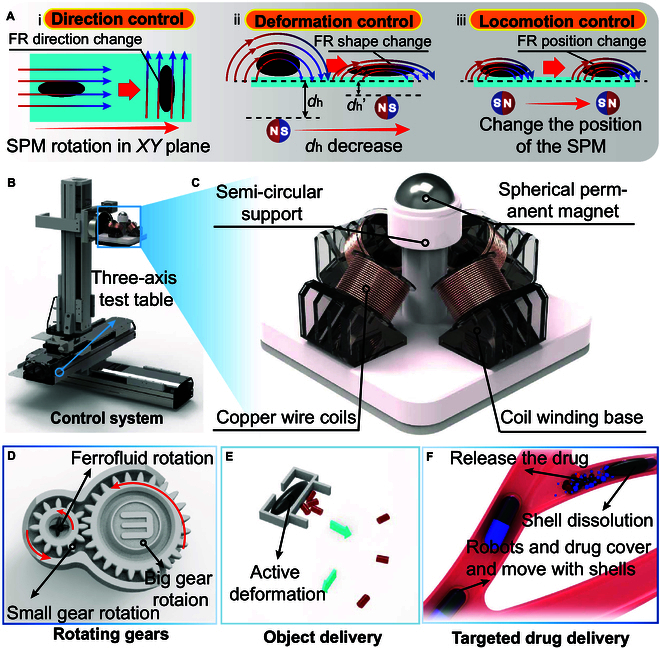
Magnetic control strategy, device, and applications of the MFR. (A) Conceptual diagram of MFR motion control. (i) Conceptual illustration of MFR directional control based on the horizontal rotation of permanent magnets. (ii) Conceptual illustration of MFR deformation control based on the vertical ascension of permanent magnets. (iii) Conceptual illustration of MFR displacement control based on the translational motion of permanent magnets. (C) Conceptual diagram of detailed components of the magnetic control device. The core components include peripheral coils and a spherical permanent magnet, where the rotation of the permanent magnet is controlled by changing the current in the coils. (D) Conceptual diagram where the MFR drives gear movement through deformation and rotation. (E) Conceptual diagram depicting the MFR driving external devices to deliver objects through deformation and displacement. (F) Conceptual diagram illustrating the MFR-based capsule performing targeted drug delivery.

To address the limitations of existing systems, this study introduces a novel magnetically actuated device based on a hybrid electromagnetic coil and permanent magnet control system. The device employs an original spherical friction strategy, utilizing weak magnetic fields generated by electromagnetic coils to regulate stronger magnetic fields produced by larger permanent magnets. By programming the 4 bottom-mounted electromagnetic coils, the permanent magnets generate enhanced magnetic fields and gradient fields to precisely control MFRs (Fig. 1B and C). Compared to conventional magnetic actuation methods, this innovative design features a more compact structure, enabling integration into small-scale mobile mechanical systems, thereby remarkably expanding the applicability of magnetic actuation devices. The device achieves low energy consumption while delivering higher and more stable magnetic fields by using electromagnetic coils to weakly actuate permanent magnets. Furthermore, the rotational motion of permanent magnets driven by electromagnetic coils enables the generation of robust external rotating magnetic fields and gradient fields. Leveraging these characteristics, the device enhances robotic motion capabilities, including precise displacement, orientation control, and shape deformation (Fig. 1A). Building on this platform, we conducted in-depth investigations into the precise motion control and deformation capabilities of MFRs, extending their functional applications. MFRs can serve as an actuation source for nonpowered mechanisms, such as driving gears and propelling carts (Fig. 1D and E). To address the inefficiencies of conventional drug delivery methods and the limitations of chemically modified nanocarriers [33], we developed a novel MFR-based capsule. This capsule leverages the magnetothermal effect induced by high-frequency magnetic fields and the magnetic field-driven actuation of the hybrid magnetic device to achieve precise drug release and targeted occlusion (Fig. 1F). The proposed electromagnetic-permanent magnet hybrid actuation system substantially broadens the functional applications of MFRs and is anticipated to advance their development in targeted medical interventions.

## Materials and Methods

### Preparation of experimental materials

**Fig. 2.**
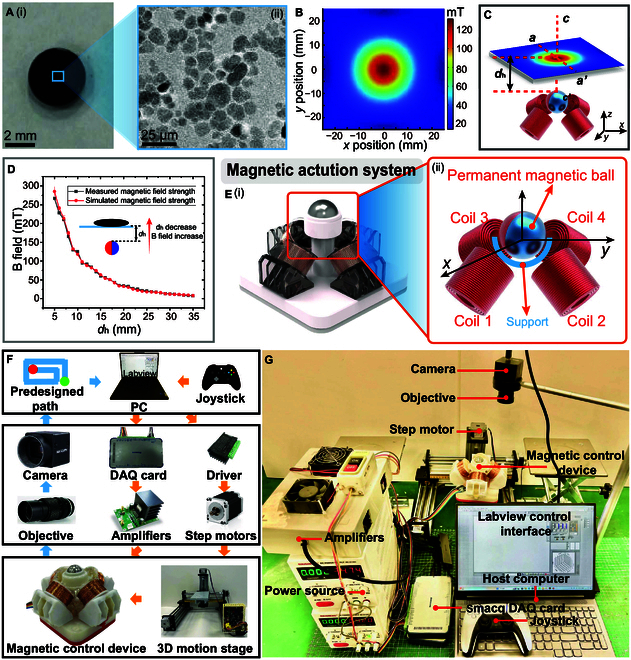
Performance of MFR and magnetic control device. (A) Macroscopic and microscopic structures of ferromagnetic fluid. (i) Macroscopic image of the ferromagnetic fluid. (ii) Scanning electron microscope (SEM) results of nanoparticles within the ferromagnetic fluid. (B) Measurement results of the magnetic flux density *B* or equivalent magnetic field $H=\frac{B}{\mu }$ within a plane 10 mm from the top of the hybrid magnetic actuation system. (C) Magnetic field distribution and coordinate definition of the hybrid magnetic actuation system, where *d*_h_ is the distance from the top of the permanent magnet to the robot’s motion plane. (D) Comparison between measured results and simulation of the magnetic field produced along the SPM polarization orientation, with subfigures showing measurements and simulation values based on decreasing *d*_h_. (E) Hybrid magnetic actuation device. (i) Complete structural diagram of the magnetic drive device; (ii) distribution and coordinate definition of permanent magnets and electromagnetic coils within the hybrid magnetic actuation system. (F) The configuration of the experimental setup. (G) Fully assembled hybrid magnetic actuation system.

We selected ferrofluid as the primary material for the MFR. Ferrofluid is a stable magnetic colloidal suspension. As illustrated in Fig. 2A, the ferrofluid comprises Fe_3_O_4_ nanoparticles with an approximate diameter of 10 nm, a carrier liquid, and a surfactant. The MFR exhibits good reconfigurability and magnetic responsiveness. Its lower viscosity and better fluidity help enhance the application efficiency of ferrofluids. The robust magnetic responsiveness also allows it to exhibit various movements and rapid transitions under an external magnetic field [21]. In addition, the silicone oil-based ferrofluid, using high-purity silicone oil as the carrier liquid and phospholipid-polyethylene glycol (phospholipid-PEG) modified Fe_3_O_4_ nanoparticles, effectively reduces toxicity, minimizes particle aggregation, and allows for slow clearance from the body, thus exhibiting good biocompatibility. The colloidal suspension comprises 25 wt%, solid content, wherein the magnetic nanoparticles constitute 40% by weight. The measured saturation magnetization (*M*_s_) is 60 ± 5 emu/g. The stabilization of the magnetic colloid is achieved through a dual-component carrier system, utilizing silicone oil as the surface-active agent and aviation kerosene as the continuous phase. For experimental purposes, we used the ferrofluid at room temperature (20°C) as the robot’s material, with a dynamic viscosity of 51 mPa·s. The resultant oil-based ferrofluid demonstrates a density (ρ) of 1.43 g/cm^3^ This optimized formulation facilitates exceptional colloidal stability and favorable rheological characteristics. The magnetic fluid exhibits remarkable long-term stability, maintaining uniform dispersion at ambient temperature (*T* = 298 K) for a duration exceeding 6 months, thereby satisfying all requisite experimental parameters. A pipette created a miniature MFR of a set volume size. The silicone oil-based ferrofluid exhibits strong hydrophobicity. Additionally, its preparation is relatively simple, and it possesses excellent deformability, allowing for stable movement in complex environments such as the vascular system, urinary system, and respiratory tract [34]. The structures used in the experiments, such as gears, delivery devices, and mazes, were fabricated using a photopolymerization 3D printer (ELEGOO Saturn 3 Ultra, ELEGOO, China). Surface cleaning was performed using a vacuum plasma cleaner (PDC-MG, MING HENG, China) to prevent adhesion between the MFR and the surface of the designed structures.

### Modeling and programming of the magnetic field

We have developed a new control device to address the limitations of traditional control strategies for MFRs, which typically have a small control range and require a time-varying magnetic field with strong gradient forces and torque. This system primarily consists of a magnetic excitation system and a locomotion drive system, offering high scalability for the overall control system. The displacement process of the hybrid magnetic actuation system is available in Movie S1 and Fig. S1.

The entire system’s magnetic field was simulated using COMSOL software, and the resulting magnetic field intensity is shown in Fig. 2B and C. Additionally, this experiment utilized a Hall sensor (TLE493D-W2B6, Infineon Technologies, Germany) to measure the magnetic field strengths along the *x*, *y*, and *z* axes. The output signals from the Hall sensor were collected via a serial port, measuring the 3 magnetic field components (*B_x_*, *B_y_*, *B_z_*) produced by the SPM and the coils. A 3-axis motorized platform traversed the workspace, and the host computer transmitted and saved the measured magnetic field data. We fixed the sliding axes in the *xy* plane and only moved the sliding axis in the *z* direction. Based on the variations in *d*_h_, we simulated and measured the magnetic field intensity generated by the permanent magnet. The magnetic field data generated by the drive device, as shown in Fig. 2D, indicate that the measured results of the magnetic excitation and locomotion drive system closely match the simulated magnetic field results. Since the magnetic actuation system is integrated with the 3-axis sliding platform, fixing the sliding axes in the *X* and *Y* directions while varying the *d*_h_ value in different magnetic field intensities is applied to the workspace, and this variation of magnetic field intensity induces deformation in the robot.

As shown in Fig. 2E, the system’s magnetic excitation system employs a combination of neodymium-iron-boron (NdFeB) spherical permanent magnets and coil electromagnets. Four orthogonally arranged coil electromagnets were symmetrically placed at the midpoints of the 4 sides of a square base, oriented at a 45° to the horizontal plane, pointing toward the center of the spherical permanent magnet. Each coil electromagnet was configured with inner and outer rings connected in parallel to increase the current and reduce resistance. Each coil had 1,500 turns, and the selected wire diameter was 0.4 mm. A semicircular support frame was installed at the focal points of the axes of the 4 coil electromagnets to restrict the position of the SPM. A steel ball with a diameter of 2.2 mm was also placed between the support and the SPM, and low-viscosity lubricating oil was applied to the contact surfaces of the 3 to minimize the friction between the SPM and the support. Compared to other magnetic drive control systems, our current work upgraded and optimized the drive control system by redesigning the support structure for the magnetic spheres. We have eliminated the direct contact between the magnetic spheres and the support structure, instead employing an original spherical–spherical friction strategy. This allows us to easily drive larger magnetic spheres (with a current diameter of 30 mm) and output greater driving force, resulting in a more flexible and powerful output. This aspect has not been explored in other magnetic drive control systems.

The locomotion drive system of the setup utilized 3 motors to drive a triaxial sliding platform, enabling the magnetic excitation device to achieve locomotion and deformation control in the *xy* plane. The triaxial sliding platform has its operational space equal to the mechanical travel range of the stage (theoretical value of 280 mm × 280 mm × 140 mm). However, due to the compact structure of the MFR, excessive expansion of the working space and imaging field of view would lead to a remarkably reduction in optical tracking resolution. Therefore, the effective operational space is constrained to the area of a circular glass dish with a diameter of 150 mm in order to balance motion control accuracy and observational stability. The validation can be found in Movie S1. The system incorporated 2 power supplies (MS-3010DS, MAISHENG, China) and 8 power amplifiers (OPA541, Texas Instruments, USA) connected to a data acquisition card with 8 channels (USB-3112, Smacq, China) to receive control inputs.

In the adaptive ferrofluid robotic system, the control system consisted of the described subsystems, as depicted in Fig. 2F and G. In this experiment, a laptop computer was used as the host system, with programming conducted in LabVIEW software for human–machine interaction and data display. Real-time monitoring of the MFR’s position was achieved using a camera, which transmits posture and other information to the control system. A high-speed data acquisition card collects and generates control signals, amplified by voltage amplifiers, transmitted to the drive coils, and used to operate the stepper motors via the drivers. In the magnetic actuation system and the 3-axis sliding platform, the magnetic control utilized 4 electromagnetic coils to generate a background-driving magnetic field, which drives the spherical NdFeB permanent magnet located along its axis, thereby driving the MFR to change shape and orientation.

### Control methods of the actuation magnetic field

This experimental setup remotely applies torque through magnetic braking to drive the MFR for rotation. The MFR is strategically positioned between the permanent magnetic sphere and the 4 electromagnetic coils at the center’s maximum magnetic field gradient point. At this position, the magnetic field denoted as ${\tilde{B}}_{i}=\left({\tilde{B}}_{i}^{x},\;{\tilde{B}}_{i}^{y},\;{\tilde{B}}_{i}^{z}\right)$ is a result of the superposition of both solid and weak driving magnetic fields. At this point, the magnetic moment of the MFR aligns with the orientation of the applied composite magnetic field, and it is subjected to the magnetic force. Furthermore, since the MFR is in a space with no electrical currents, the magnetic field created can be described using Maxwell’s equations. The expression of the force $F_{m}$ and torque $\tau_{m}$ of MFR with a magnetic moment m in the magnetic field B can be simplified as [35]:

$$\begin{array}{c} \tau_{m}=m\times B=\left[\begin{array}{ccc} 0 & B_{z} & -B_{y}\\ -B_{z} & 0 & B_{x}\\ B_{y} & -B_{x} & 0 \end{array}\right]\;\left[\begin{array}{c} m_{x}\\ m_{y}\\ m_{z} \end{array}\right] \end{array}$$ (1)

$$\begin{array}{c} F_{m}=\left[\begin{array}{ccc} \frac{\partial B_{x}}{\partial x} & \frac{\partial B_{x}}{\partial y} & \frac{\partial B_{x}}{\partial z}\\ \frac{\partial B_{x}}{\partial y} & \frac{\partial B_{y}}{\partial y} & \frac{\partial B_{y}}{\partial z}\\ \frac{\partial B_{x}}{\partial z} & \frac{\partial B_{y}}{\partial z} & -\left(\frac{\partial B_{x}}{\partial x}+\frac{\partial B_{y}}{\partial y}\right) \end{array}\right]\;\left[\begin{array}{c} m_{x}\\ m_{y}\\ m_{z} \end{array}\right] \end{array}$$ (2)

The magnetic control device designed in this paper combines a weak driving magnetic field generated by a 4-axis electromagnetic coil with a strong driving magnetic field from a permanent magnet sphere, enabling the MFR to align with the magnetic field direction automatically. In the device we designed, the position of the permanent magnet is fixed and exhibits isotropy in the magnetic field. The magnetic field generated by the electromagnetic coils only needs to drive the permanent magnet for rotational direction control. Therefore, we only need to consider the relationship between the currents in the coils and the magnetic torque. Assuming that the magnetic field vector generated by multiple coils at MFR position P is: ${\tilde{B}}_{i}=\left({\tilde{B}}_{x}^{i},\;{\tilde{B}}_{y}^{i},\;{\tilde{B}}_{z}^{i}\right)$. The contribution matrix $\beta \left(P\right)$ of the magnetic coil assembly in this system can be composed of multiple $\tilde{B}$ produced by each coil. Considering the linear superposition of the system’s net magnetic flux density, the magnetic field vector at MFR position *P* is obtained by multiplying the electromagnetic coil’s contribution matrix with the coil current matrix [36]:

$$B\left(P\right)=\beta \left(P\right)\left[\begin{array}{c} i_{1}\\ \vdots \\ i_{n} \end{array}\right]$$ (3)

In this system, we set the center of the permanent magnet as the origin. $\beta \left(P\right)$ is the contribution matrix of each coil at the original point. By determining the contribution matrix, we can solve for the currents in each coil based on the linear relationship between the magnetic field and the coils. This characteristic of the device, β(P), can thus be simplified as [37]:

$$\beta \left(P\right)=a\;\left[\begin{array}{cccc} 1 & -1 & -1 & 1\\ 1 & 1 & -1 & -1\\ -1 & -1 & -1 & -1 \end{array}\right]$$ (4)

The proportionality coefficient a is related to the coil’s dimensions, number of turns, and other design parameters. Since the coils are symmetrically arranged and their axes intersect at the origin, it can be assumed that the magnetic flux density at the origin is equal along the *x*, *y*, and *z* axes. Therefore, the proportionality coefficient a is the same for all coils, and the value of a does not affect the calculation results of the currents in the coils. In addition, due to the composition of its nanoparticle constituents, the MFR can exhibit behavior similar to that of a dipole under the influence of an external magnetic field. To further establish a composite magnetic field model that accounts for the impact of permanent magnets and electromagnetic coils on the behavior of the MFR, this paper has developed a corresponding magnetic dipole model to describe the response of the MFR in external magnetic fields.

$$B\left(m\cdot r\right)=\frac{\mu_{0}}{4\pi }\left(\frac{3m\cdot r}{r^{5}}r-\frac{m}{r^{3}}\right)$$ (5)

where B is the magnetic flux density produced by the dipole, $\mu_{0}=4\times 10^{-7}$ is the magnetic permeability of air, r is the vector pointing from the center of the magnet to the target point $P\left(r,\;\theta \right)$, and m is the magnetic moment of the MFR.

### Modeling and programing of the motion control

The MFR’s dynamic system exhibits coupled multiphysical interactions during locomotion. This study employs a lumped-parameter modeling framework that neglects interparticle interactions within the ferrofluid while considering 4 primary governing forces: magnetic force *F*_m_ from permanent magnets, net gravitational force *F*_G_, robot-boundary interaction force *F*_b_, and viscous resistance *F*_d_. Under the constraints of the scallop theorem, the low Reynolds number hydrodynamic environment achieves quasi-static equilibrium through viscous resistance $F_{d}=6\pi r_{0}\mu V$, where V represents the MFR’s velocity vector and μ represents the dynamic viscosity of water. The mechanical model of MFR is formulated as follows: $F_{m}\left(i,\;j\right)+F_{G}+F_{b}=F_{d}$. This formulation characterizes the quasi-static motion in viscous dissipation-dominated regimes. The net gravitational force $F_{G}=\frac{4\pi r_{0}^{3}}{3}g\Delta \rho$, where Δρ represents the density difference between ferrofluid and water. $F_{b}=-K\left(z_{i}-r_{0}\right)$ represents the repulsive force generated during MFR substrate collision, where $z_{i}$ represents the height of the microrobot from the substrate and K is the stiffness coefficient of the MFR.

The magnetically actuated microrobot exhibits a critical minimum driving frequency *f*_min_ during its transition from static to dynamic states. This parameter quantifies the threshold excitation required to overcome static friction resistance, viscous dissipation, Brownian perturbations, and environmental disturbances. Synchronized motion between the MFR and magnetic actuator occurs exclusively when the external magnetic driving frequency exceeds this critical value. Crucially, during sustained locomotion, the ferrofluid’s self-locking effect establishes magnetic force dominance, enabling justified neglect of secondary effects, including inertial forces and viscous dissipation. Building upon this physical rationale, the MFR’s locomotion can be characterized as a first-order dynamic system. Guided by this theoretical foundation, we developed a synergistic control strategy. For the vision module, the system adopts an optical–magnetic cooperative perception framework, which is divided into 3 layers: In the image acquisition layer, a charge-coupled device (CCD) camera is positioned directly above the magnetic control system to synchronously capture the planar motion video stream of the robot along with its *xy* coordinates. In the data transmission layer, the captured images are transmitted in real time to the upper-level control system as a sequence of frames. In the image processing layer, based on the difference in light absorption between the magnetic fluid in the MFR and the surrounding environment, an adaptive RGB threshold is set. Through binarization processing, the MFR region is mapped as a black connected domain while the background remains white. On the binarized image, the meanshift algorithm is introduced for target tracking. During the tracking process, a centroid localization approach is adopted. Specifically, after initializing the search window, an iterative calculation of the pixel density gradient within the window is performed, which converges to the centroid of the robot. This method effectively adapts to the remarkable deformations experienced by the MFR during motion while maintaining accurate tracking. This vision-based method dynamically couples optical perception with magnetic control commands, ensuring that the system meets real-time performance requirements. The specific control process can be seen in Fig. S2. The controller’s principal function resides in real-time computation of optimal current configurations for each driving coil, which is essential for generating precise magnetic gradient fields governed by electromagnetic spatiotemporal characteristics. By controlling the coil currents in Eq. (3), the MFR’s motion strictly adheres to the dynamic principles established in Eqs. (6) and (7). Specifically, Eq. (6) constitutes a first-order linearized dynamic model that quantitatively characterizes the velocity profile of the MFR within the *xy* plane.

$$\left\{\begin{array}{cc} x\left(t\right) & =v_{1}\left(w\left(t\right)\right)+b\left(t\right)\\ y\left(t\right) & =v_{2}\left(w\left(t\right)\right)+b\left(t\right)\\ \phi \left(t\right) & \;=\Theta \left(t\right)+b\left(t\right) \end{array}\right.$$ (6)

where $v_{1},v_{2}$ is the velocity components of MFR in the *xy* plane. $w\left(t\right)$ represents the angular velocity at time *t*. $\phi \left(t\right)$ are the rotational angular velocities of the MFR and the magnetic field, respectively. $b\left(t\right)$ represents random disturbances and noise in the liquid environment. After acquiring the real-time position data of the MFR, the control unit calculates its positional deviation from the preset waypoints and generates forward driving commands based on the predefined kinematic control laws. The mathematical formulation follows the mechanism outlined below.

$$v=k_{p}\text{min}\left(\sqrt{e_{x}^{2}+e_{y}^{2}}+f_{\min},\;f_{\max}\right)$$ (7)

$$\begin{array}{c} \Delta \theta =\left\{\begin{array}{cc} \text{arctan}\left(\frac{e_{y}}{e_{x}}\right) & \text{if}\;\text{arctan}\left(\frac{e_{y}}{e_{x}}\right)<20^{\circ }\\ 20^{\circ } & \text{if}\;\text{arctan}\left(\frac{e_{y}}{e_{x}}\right)\geq 20^{\circ } \end{array}\right. \end{array}$$ (8)

where *k*_p_ represents the ratio of the MFR’s velocity to the driving frequency, which governs the movement speed of the MFR. $e_{x}$ and $e_{y}$, respectively, represent the distance error components on the *x* axis and *y* axis between the current position and the expected position of the MFR. $\left(x_{c},\;y_{c}\right)$ is the current location and $\left(x_{r},\;y_{r}\right)$ is the desired location of the MFR. $e_{x}=x_{c}-x_{r},e_{y}=y_{c}-y_{r}$, $f_{\max}$ is the maximum frequency at which the magnetic actuation system can synchronize the MFR with the rotating magnetic field.

## Results

### Locomotion, orientation, and deformation control experiments of MFRs

Based on the newly designed hybrid magnetic actuation system and the corresponding closed-loop visual control strategy, the system decouples the trajectory and posture control of the MFR. The flowchart of the control system is shown in Fig. S2. Compared to magnetic control devices such as Helmholtz coils and electromagnetic coil arrays, these systems exhibit notable control limitations. Ferrofluid robots cannot achieve decoupled control of direction, deformation, and locomotion in Helmholtz coils. For electromagnetic coil array-based magnetic control devices, there are marked limitations in the DOF for controlling the robot’s orientation. The MFR decoupled control optimizes its unique dynamics and control requirements by managing position and orientation control separately. While maintaining its original locomotion capabilities, it allows for more complex and precise MFR-driven control, reducing the complexity of control and enhancing precision.

**Fig. 3.**
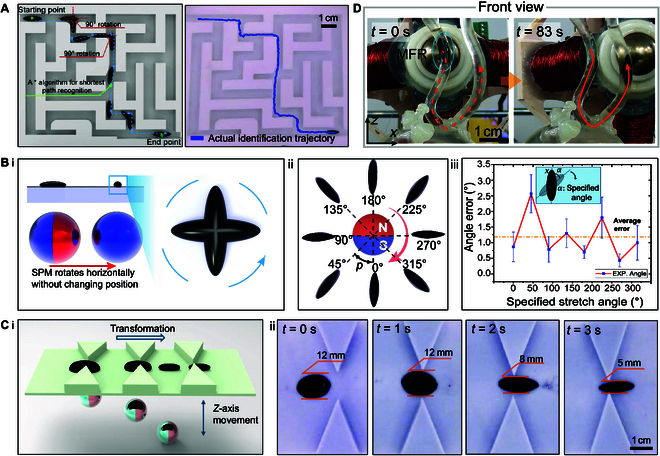
Locomotion, orientation, and deformation control experiments of MFR. (A) Actual experimental motion trajectory and motion simulation results of MFR in a complex maze. (B) Under the effect of magnetic field and magnetic torque, MFR’s controllable steering. (i) MFR controllable steering simulation. (ii) Controllable steering experimental results. (iii) Stretch angle errors of the MFR at specific positions. (C) Under deformation and magnetic field gradient forces, MFR’s controllable deformation through narrow passages. (i) MFR controllable deformation simulation experiment. (ii) Experimental results of controllable deformation through narrow passages. (D) The robust locomotion capability of the MFR within a simulated 3D vascular environment.

As shown in Fig. S3 and Movie S2, the MFR moves within a circular glass petri dish with a diameter of 150 mm. The overall trajectory forms a regular parallelogram with a height and base of 40 mm. The final positioning error is 0.1657 mm, which is relatively small compared to the overall size of the device and the trajectory. The standard deviation obtained by fitting a Gaussian curve was 0.065668 (Fig. S3), verifying the feasibility of the device and the closed-loop control strategy. As shown in Fig. 3A and Movie S3, based on the closed-loop visual control strategy, the orientation and position control of the MFR were integrated, and a complex maze model was designed. During the experiment, the A* algorithm was used for path planning, allowing the MFR to execute synchronized turning and navigate narrow passages, reaching the endpoint via the shortest path. As shown in Fig. 3B, by adjusting the orientation and intensity of the compound gradient field and the rotational magnetic field generated by the drive system, forces and torques are remotely applied in a magnetic braking manner, customizing the magnetic field by adjusting the orientation of the rotational field. This precise control over the stretching orientation of the MFR, exploring different angles of MFR extension, results in excellent orientational control performance, with an average error of 1.46° and a maximum error of 3.26°. It is important to emphasize that when controlling the robot’s direction, some deformation occurs. This is due to a combination of errors in the assembly of the magnetic control device, minor magnetic field distortions caused by the experimental environment, and the imperfect flatness of the substrate. As shown in Fig. 3C and Movie S4, by independently controlling the *z*-axis displacement within the triaxial sliding stage, the distance between the magnetic actuation system and the MFR is controlled, adjusting the gradient of the compound gradient magnetic field. Some magnetic particles move toward the orientation of gradient change, enabling the MFR to stretch and deform to pass through narrow channels. The deformability of the MFR is represented by the ratio of its primary to minor axes, with specific data presented in Fig. S4. As shown in the figure, the maximum stretch ratio reaches 3.6 at a distance of approximately 16 mm. This variable reconfigurability enhances the feasibility of MFRs in complex internal human environments, such as bronchial tubes, bronchi, and bile ducts. In those cases, if an MFR navigates through a narrow passage without adequate deformation control, it risks becoming obstructed, especially in confined tubular spaces. Furthermore, we conducted 3D locomotion experiments within a simulated vascular environment, as demonstrated in Fig. 3D and Movie S5. The biomimetic vascular phantom was fabricated via stereolithography apparatus, with its physiological environment simulated by tuning the rheological properties of a glycerol–water mixture. The results indicate that the hybrid magnetic actuation system enables MFR to achieve stable motion within this complex 3D vascular topography. This advancement significantly broadens the potential of MFR for biomedical applications, particularly in navigating intricate biological luminal structures.

### Combined motion control experiments of MFRs

**Fig. 4.**
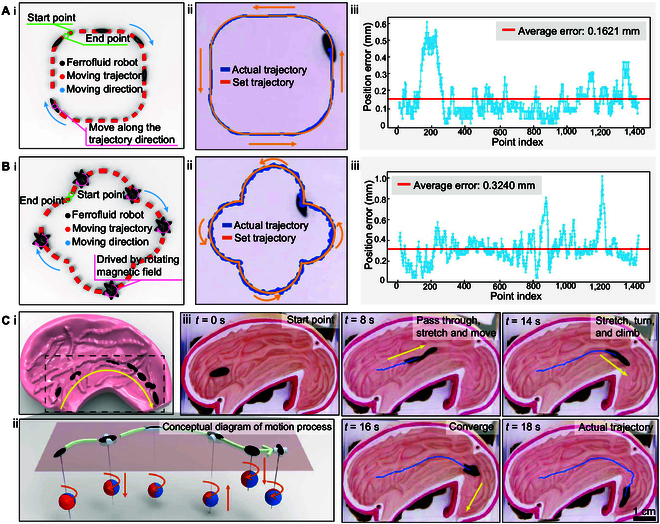
Combined motion control experiments of MFR. (A) MFR moving along the rounded square trajectory. (i) Simulation of MFR trajectory, orientation aligned tangentially to the rounded square path. (ii) Path tracking results of the rounded trajectory. (iii) Rounded square trajectory tracking error. (B) MFR moving along the flower-shaped trajectory formed by 4 semicircles. (i) Simulation of MFR trajectory in self-rotation. (ii) Path tracking results of the floral-shaped trajectory. (iii) Floral-shaped trajectory tracking error. (C) Under the combined control of gradient and magnetic torque, an experiment simulating human stomach fold passage. (i) Simulation of MFR magnetic field combined control. (ii) A conceptual diagram illustrating the complete motion process under multidimensional coordinated control of direction, deformation, and displacement in a simulated gastric environment, depicting the positional variations and rotational dynamics of a magnetic sphere. (iii) Experimental results of navigating, shuttling, and converging within a simulated stomach.

Based on the stability of independent control of MFR in locomotion, direction, and deformation, we attempted to integrate these abilities to validate the motion robustness of MFR under combined control. As illustrated in Fig. 4A and B and Movies S6 and S7, 2 types of orientational motion experiments were conducted. As shown in Figs. 4A(i) and (ii), an MFR moves along a rounded square path, with the orientation of the MFR stretching continuously tangential to the motion trajectory. In Figs. 4B(i) and (ii), the MFR follows a floral-shaped trajectory (with each side of a square serving as the diameter for outward circles). At the same time, the magnetic field is set to a circular pattern, generating a constant rotational force, causing the MFR to rotate around its center. The error line graphs for these trajectories, shown in Figs. 4A(iii) and B(iii), present an average error distribution of 0.1621 and 0.3240 mm. Conventional magnetic control systems (e.g., Helmholtz coils and distributed electromagnetic arrays) exhibit substantial trajectory tracking errors (Δe=0.3to0.9mm) due to steep magnetic field gradients at workspace boundaries. In contrast, our proposed electromagnetic-permanent magnet hybrid actuation system achieves remarkably reduced trajectory errors. The Gaussian curve fitting of the trajectory errors revealed standard deviations of 0.10978 and 0.14823 (Figs. S5 and S6), indicating high control precision of the MFR after decoupling its pose and integrating closed-loop control strategies, suitable for precise position and orientation control in complex environments and tasks. To further validate the superior complex control capabilities of the magnetic actuation system, as illustrated in Fig. 4C(i) and Movie S8, we designed a motion experiment demonstrating flexible navigation through folds to reach target locations in a human stomach model. The conceptual diagram in Fig. 4C(ii) comprehensively displays the positional relationship between the MFR and the orientation of the permanent magnet within the magnetic actuation system. As shown in Fig. 4C(iii), at 8 s, the MFR successfully navigated through gastric folds and narrow channels through synchronized control of posture and deformation. By 16 s, through elevation of the magnetic actuation system, the magnetic field strength was enhanced at the MFR’s location, enabling precise convergence of the MFR at the position. These experimental results demonstrate that MFR provides a noninvasive and operationally flexible tool, offering a viable solution for the precise delivery of drugs to specific lesion areas within the stomach.

### Motion-enabling mechanisms for passive bodies in complex environments

**Fig. 5.**
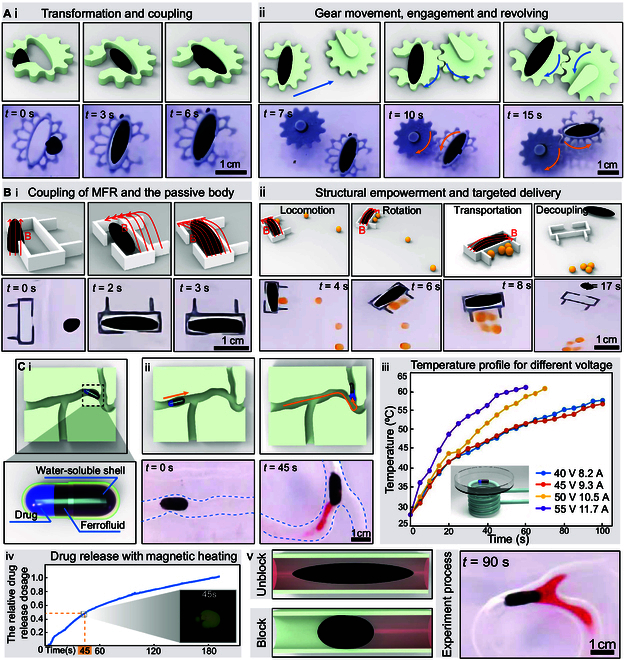
Combined control experiments of MFR in complex environments. (A) MFR is a power pack drive gear mechanical structure. (i) MFR coupling in locomotion with the gear mechanism under the influence of gradient and torque. (ii) MFR driving gear engagement and revolving. (B) MFR coupled delivery mechanism experiments for transporting items. (i) MFR couples with a passive body. (ii) MFR drives the passive body for drug delivery. (C) MFR empowered a capsule for drug-targeted delivery and selective blockage in simulated vascular environment. (i) Drug capsule driven by MFR reaches a specific location for drug delivery, showing experimental results. (ii) The impact of high-frequency magnetic field voltage on heating is shown in a line chart. (iii) Schematic of the capsule structure. (iv) The drug release profile of the capsule accelerated by the high-frequency electromagnetic heating strategy. The drug dose at complete release is normalized to 1. The subfigure is derived by subtracting the first frame from subsequent frames of the recorded video. (v) Conceptual diagram and experiment process of MFR selective occlusion in simulated vascular environment.

Fig. 5A and Movie S9 demonstrate that the MFR can be designed as a simple mechanical structure, serving as a power pack to empower mechanical structures and expand the applications of MFR. A gear mechanism with coupling features was designed to validate the empowerment effect of MFR on mechanical structures. Through the power chamber design of the gear mechanism and external retention channels, the MFR can couple with the gear mechanism’s motion control, thereby driving the transmission between multiple gears. As shown in Fig. 5A(i), during the coupling stage, the MFR enters the gear power chamber through the external channel in an unextended state. Through the rotational and compound gradient magnetic fields generated by the magnetic actuation systems, the MFR stretches and fills the gear mechanism power chamber, achieving coupled pose control. As shown in Fig. 5A(ii), in the motion control and operation section, through the coupled pose relationship between the MFR and the mechanism, by synchronously controlling the magnetic actuation system, the rotation and displacement of the gears are decoupled and controlled, enhancing gear control precision and functionality. As shown in Fig. 5A, at *t* = 3 to 6 s, the MFR couples with the gear drive mechanism; at *t* = 7 to 15 s, the MFR first drives the gear to mesh with the central gear, and after meshing with the gear, through the decoupled control of the position and extension rotation of the MFR, it synchronously controls the position and rotation of the external gear. The angular velocity profile synchronizes with the temporal derivative of the MFR’s extensional rotation, demonstrating orbital rotation about the central gear’s principal axis while actively driving the central gear’s angular displacement. This mechanism establishes kinematic coupling between the dual-gear system through coordinated torque transmission. In the experiment, the driving gear weighs 5 g, and the driven gear weighs 10 g. The friction coefficient between the gears and water $\mu_{1}=0.15$, and the friction coefficient between the gears themselves $\mu_{2}=0.4$. To determine the final force needed to rotate the gear, we first need to calculate the moment of inertia of each gear. According to the formula for the moment of inertia: $I=\frac{1}{2}\mathrm{mr}$, where I is the moment of inertia of the gear, m is the mass of the gear, and r is the radius. The final calculation gives the total moment of inertia as $1\times 10^{-6}$. To rotate both gears, it is necessary to overcome inertia, water friction, and friction between the gears. Therefore, the total torque $\tau_{\text{total}}$ can be calculated using the following dynamic equation:

$$\tau =\frac{\left(I_{\text{total}}\cdot \alpha +\tau_{\text{water}}\right)}{\left(1-\mu_{2}\right)}$$ (9)

where α is the angular acceleration and $\tau_{\text{water}}$ is the frictional torque between the gear and water, which is calculated as $3.69\times 10^{-4}$. According to the formula for tangential force: $F=\frac{\tau }{r}$, where F is the tangential force required to rotate the gear, the calculation yields that the tangential force required to drive the main gear is 36.9 mN. Additionally, the tangential force required to drive the driven gear is 22.1 mN. In addition, using formulas $m=\rho \cdot V\cdot M_{s}$, τ=m×B, and $F=\frac{\tau }{r}$, where m is the magnetic dipole moment of the MFR, $\rho =1.43\;g/{\text{cm}}^{3}$ is the density of the MFR, $V=0.5027\;{\text{cm}}^{3}$ is the total volume of the MFR, $M_{s}=60\;\text{emu}/g$ is the measured saturation magnetization of the MFR, B is the magnetic field strength, r is the lever arm, and τ and F are the torque and force, respectively, obtained as the final solutions. It is calculated that the magnetic droplet robot can generate a maximum torque of about 0.0129 Nm and can produce a torque force of 1.73 N. Therefore, we can see that through this magnetic control device, the robot can apply forces far exceeding those required to rotate the gears. The MFR synchronously controls its orientation, position, and deformation, acting as a power pack to empower mechanical structures. It can further be used to drive complex mechanical structures.

Building on the experimental result, which demonstrates the MFR’s torque generation in a passive gear assembly and the substantial gradient force provided by the magnetic actuation system, as illustrated in Fig. 5B and Movie S10, we further investigate the functionality of the MFR as a power unit in a targeted material delivery device. The experiment designed a miniature delivery device comprising a material-loading chamber and a power chamber. The material-loading chamber, a smaller rectangular structure with a length of 8 mm, is designed to collect and transport drugs. The power chamber, a larger open rectangular structure measuring 11 mm, accommodates the MFR as the power unit to drive the device. In this setup, the MFR enters the delivery device from the external environment and undergoes deformation to control the device’s position and orientation, collecting and transporting simulated drug particles to a designated location. Microspheres with a diameter of 4 mm and a mass of 43 mg were used to simulate the drug payload. As shown in Fig. 5B(i), during the deformation stage, the initial distance (*d*_h_) between the permanent magnet and the MFR was set to 17 mm, with no electromagnetic field applied, allowing the MFR to maintain a conical structure. This configuration minimized axial length, leveraging the MFR’s superior deformation capability to enter the power chamber. Subsequently, an electromagnetic field was applied to alter the axial orientation of the permanent magnet, inducing MFR deformation. At this stage, the MFR achieved a deformation ratio (aspect ratio) of 3.3 under a magnetic field strength of 25 mT generated by the magnetic control system. However, the MFR did not fully conform to the power chamber’s geometry, resulting in partial loss of thrust and torque. To optimize performance, the *d*_h_ value was reduced to 13 mm, lowering the deformation ratio to 2.3, while the magnetic actuation system increased the field strength to 48 mT. This adjustment enabled the MFR’s geometry to closely match that of the power chamber, effectively empowering the passive mechanical component. As depicted in Fig. 5B(ii), during the locomotion stage, the external magnetic field strength remained at 48 mT, consistent with the deformation stage, maintaining the *d*_h_ value at 13 mm. The MFR achieved an average velocity of 14.48 mm/s throughout the process and delivered a 172-mg payload over a distance of approximately 90 mm in 6 s. Finally, as shown in Fig. 5B(ii), at 17 s, the MFR successfully displaced the delivery device from the collection site to the target site and achieved decoupling from the device. This experiment demonstrates that, by empowering the passive mechanical component and leveraging the rapid response of magnetic control, the MFR enables targeted material delivery with higher precision and greater payload capacity compared to conventional magnetic actuation systems. Furthermore, the MFR’s ability to provide substantial driving force to the passive mechanical component allows for the effective expulsion of foreign objects from the body, such as button batteries or plastic toys accidentally ingested by infants. This highlights the MFR’s potential for both therapeutic and safety-related applications.

The functionality of the MFR in empowering passive bodies also provides novel avenues for targeted therapeutic applications within simulated vascular environment. To this end, as depicted in Fig. 5C(i), we developed a magnetic capsule robot encapsulated in gelatin, loaded with a simulated drug (pigment), and powered by the MFR’s high magnetic responsiveness to propel the capsule to a target site. A 130 mm × 130 mm vascular model was employed to simulate the complex human vascular environment, characterized by intricate curvature and varying cross-sectional areas. The motion dynamics, as illustrated in Fig. 5C(ii) and Movie S11, demonstrate that the MFR, functioning as the power unit, employs precise position and orientation control to steer and navigate the capsule through curved channels synchronously. This enables stable delivery of the capsule to the target site, followed by the release of the simulated drug. The capsule’s translational velocity, as shown in Fig. S7, exhibits a functional dependence on both the *d*_h_ and the MFR’s mass fraction within the capsule. Smaller *d*_h_ values and higher MFR mass fractions result in increased capsule velocities, reaching up to 13 mm/s. This is attributed to the enhanced magnetic gradient force exerted by the actuation system at smaller *d*_h_ values and the greater thrust generated by a higher MFR mass fraction under magnetic excitation. However, it is noteworthy that excessively small *d*_h_ values may induce MFR division, reducing the overall torque applied to the capsule during directional changes. To balance high translational velocity with stable steering, we optimized the *d*_h_ value to 6 mm in the experiments. Upon reaching the target site, the gelatin capsule dissolves, releasing the drug payload. This approach underscores the MFR’s potential for precise, magnetically controlled drug delivery in complex vascular environments. To accelerate the dissolution of the capsule shell, an additional set of electromagnetic coils was integrated to enable high-frequency magnetic heating, with the temperature depicted in Fig. 5C(iii). This mechanism exploits the ability of magnetic nanoparticles to undergo magnetic moment reorientation under a high-frequency alternating electromagnetic field. During this process, the nanoparticles generate heat through frictional interactions with the surrounding medium due to their physical rotation. Additionally, the internal reorientation of magnetic moments overcomes energy barriers, further contributing to heat production. Leveraging these mechanisms, the heat generated by the MFR enables localized heating of the target site’s surrounding environment, significantly accelerating the dissolution of the capsule shell and facilitating precise temporal control of targeted drug release. To quantify the enhancement of drug release efficiency by the magnetic heating strategy, a drug release time curve was introduced to characterize the release process under magnetic heating conditions. As shown in Fig. 5C(iv), when the high-frequency electromagnetic coils heat the system to approximately 45°C, the capsule releases the simulated drug at a significantly fast rate, achieving 50% payload releases within 45 s. Furthermore, a subplot in Fig. 5C(iv) presents optical images of the capsule’s drug release at 45 s, confirming the accuracy of these findings. Notably, although this temperature is slightly above human body temperature, it remains below the threshold for causing burns or other tissue damage. As illustrated in Fig. 5C(ii), at *t* = 45 s, the capsule shell dissolves, and the simulated drug is gradually released at the intersection of 3 channels. Importantly, the capsule shell is a solid structure composed of water-based materials such as gelatin and glycerol. In contrast, the MFR is a liquid formulated from oil-based materials, while the simulated drug is an aqueous solution. Consequently, these components remain immiscible, forming a water-in-oil structure within the capsule. This structural arrangement ensures that the MFR maintains its integrity and independence following drug release.

Leveraging this capability, we propose a selective occlusion strategy utilizing the MFR to temporarily occlude healthy vessels upstream of a lesion, thereby creating a localized fluid isolation zone that confines drug release to the vicinity of the target lesion. In contrast, conventional chemotherapeutic approaches, when administered in the human vascular system, are subject to fluid dynamic dispersion, leading to nonspecific drug diffusion, with up to 62% of the administered dose inadvertently distributed to healthy tissues [38,39]. As demonstrated in Fig. 5C(v) and Movie S11, the dissolution of the gelatin capsule shell does not impede the MFR’s mobility. By modulating the orientation and magnitude of the composite gradient magnetic field, in conjunction with the rotating magnetic field generated by the actuation system, the MFR’s trajectory can be precisely controlled. Here, we maintain the MFR’s position at the target site by precisely modulating the deformation rate through adjustments to the *d*_h_ value, enabling rapid transitions between occluded and unobstructed flow states. Specifically, as shown in Movie S11, increasing the *d*_h_ value from 6 to 14 mm enhances the MFR’s deformation ratio to 3. At *t* = 90 s, during drug release, the MFR selectively blocks the left channel, allowing the simulated drug to diffuse along the remaining channels while preventing entry into the blocked channel. This approach facilitates targeted drug delivery directly to the lesion site, maximizing therapeutic efficacy while minimizing adverse effects on healthy tissues.

## Discussion

This study presents a hybrid magnetic actuation system that extends the functional capabilities of MFR through synergistic multiphysical interactions. The innovative system features a compact hybrid-drive architecture integrating a triaxial stage with the magnetic actuation system, overcoming the energy efficiency and multimodal control limitations of conventional magnetic actuation systems. Leveraging an original sphere-to-sphere frictional coupling strategy, the system achieves high-torque output with reduced energy consumption, enabling precise regulation of MFRs’ locomotion, orientation, and morphological reconfiguration. By dynamically coupling MFRs with passive mechanical assemblies, we established a novel magneto-mechanical transmission system capable of microscale gear meshing transmission and targeted material transport. In addition, experimental results demonstrate unique biomedical advantages in targeted drug transport (velocity > 5 mm/s): leveraging the precise control capabilities of a composite magnetic actuation system and the robust deformation ability of MFR, ferrofluid drug capsules can be selectively dissolved at specific sites to release drugs, while MFR can selectively occlude blood vessels to enhance the accumulation of chemotherapeutic agents near the lesion. The hybrid magnetic actuation system developed in this study demonstrates significant advantages in terms of compactness: the device has overall dimensions of 14 × 14 × 10 cm^3^ and holds potential for further miniaturization. In contrast, conventional magnetically controlled systems (such as Helmholtz coil or distributed electromagnetic arrays) are typically constrained by a relatively small effective workspace [40,41]. While the system’s centralized control architecture theoretically enables workspace scalability through mobile mechanical structure reconfiguration, the current study constrains operational regions to standard 180-mm-diameter petri dishes.

Building on the hybrid magnetic actuation system, current research has successfully expanded the locomotion capabilities and application scenarios of the MFR, notably by endowing passive objects with kinetic functionality. However, existing studies have primarily focused on exploring and validating the MFR’s functional attributes, leaving the impact of the dynamic human physiological environment on its motion performance underexplored. For instance, the significant viscosity variations within the human gastrointestinal tract necessitate further investigation into the MFR’s locomotion performance in variable-viscosity environments [42,43]. Similarly, variations in vascular diameter within the circulatory system induce substantial changes in blood flow pressure, resulting in significant fluctuations in fluid shear forces acting on the MFR. The MFR’s motion capabilities in such complex environments, characterized by variable shear forces and flow velocities, require further evaluation [44]. Notably, the current visual module, which relies on a CCD camera, is constrained by experimental limitations and is unsuitable for clinical applications. To address this, future research will integrate ultrasound probes to assess the MFR’s tracking performance under acoustic shadowing and Doppler effects in biological tissues. Additionally, we plan to incorporate radiopaque particles into the MFR and employ x-ray imaging systems to enable real-time visualization of enclosed anatomical structures in minimally invasive clinical applications [45].

## Data Availability

All data needed to evaluate the conclusions in the paper are present in the paper and the Supplementary Materials. Additional data related to this paper may be requested from the authors.
