# Supplementary material for: Adaptive Ferrofluidic Robotic System with Passive Component Activation Capabilities
Source: Cyborg Bionic Syst. 2025 Jun 24;6:0300. doi: 10.34133/cbsystems.0300 (PMC12187217; doi:10.34133/cbsystems.0300)
Supplement: Supplementary 1 — Figs. S1 to S7 Movies S1 to S11 [file cbsystems.0300.f1.zip › Supplementary Figure 1-7.pdf]

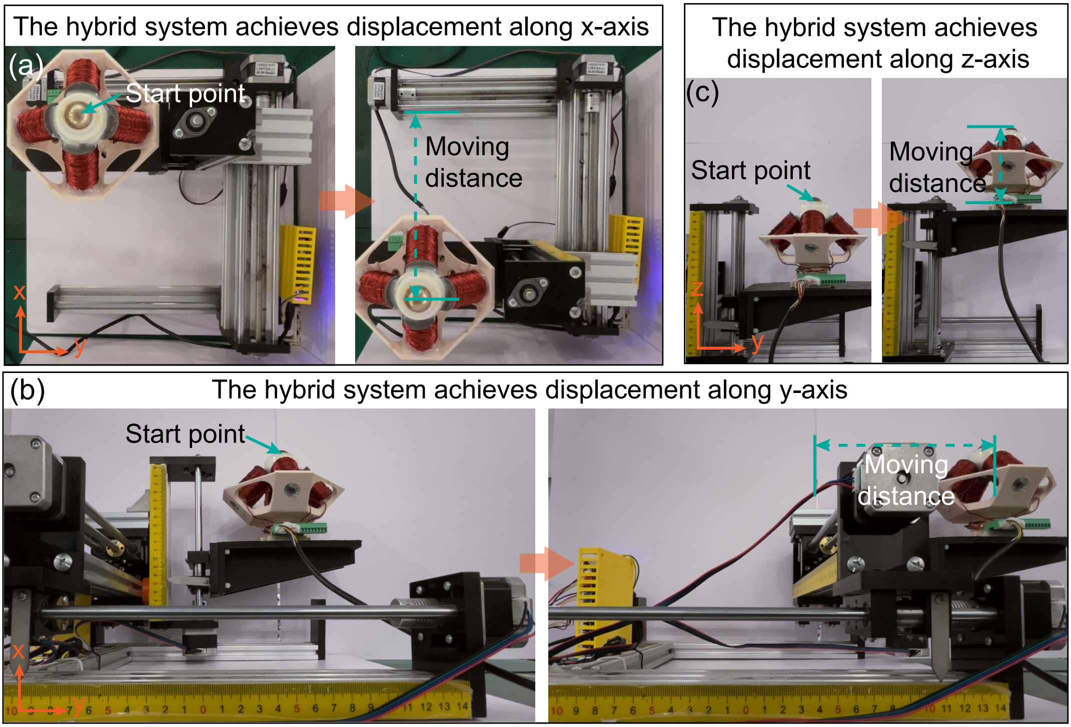
The hybrid system achieves displacement along x-axis
(a)
Start point
Moving
distance
x
y
The hybrid system achieves
displacement along z-axis
(c)
Start point
Moving
distance
z
y
(b)
The hybrid system achieves displacement along y-axis
Start point
Moving
distance
x
y

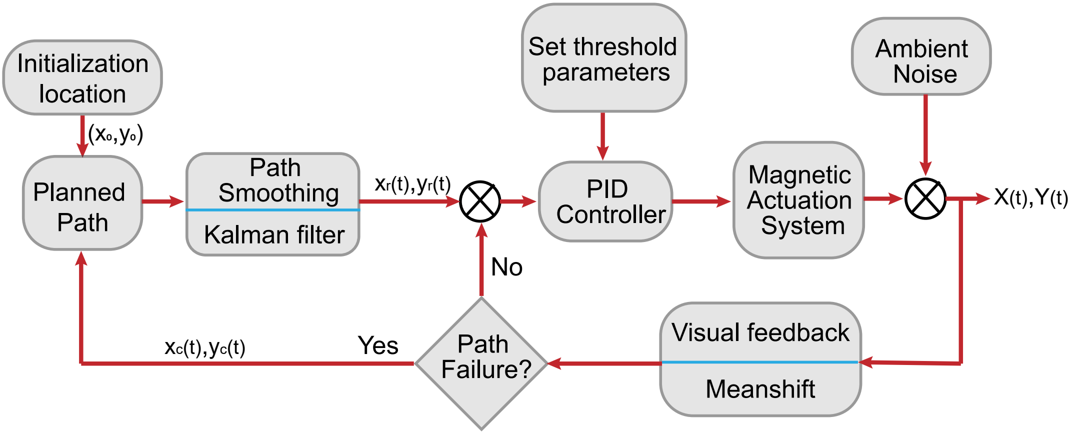

Initialization location
(xₒ,yₒ)
Planned Path
Path Smoothing
Kalman filter
xr(t),yr(t)
Set threshold parameters
PID Controller
Magnetic Actuation System
Ambient Noise
X(t),Y(t)
No
Visual feedback
Meanshift
Path Failure?
Yes
xc(t),yc(t)

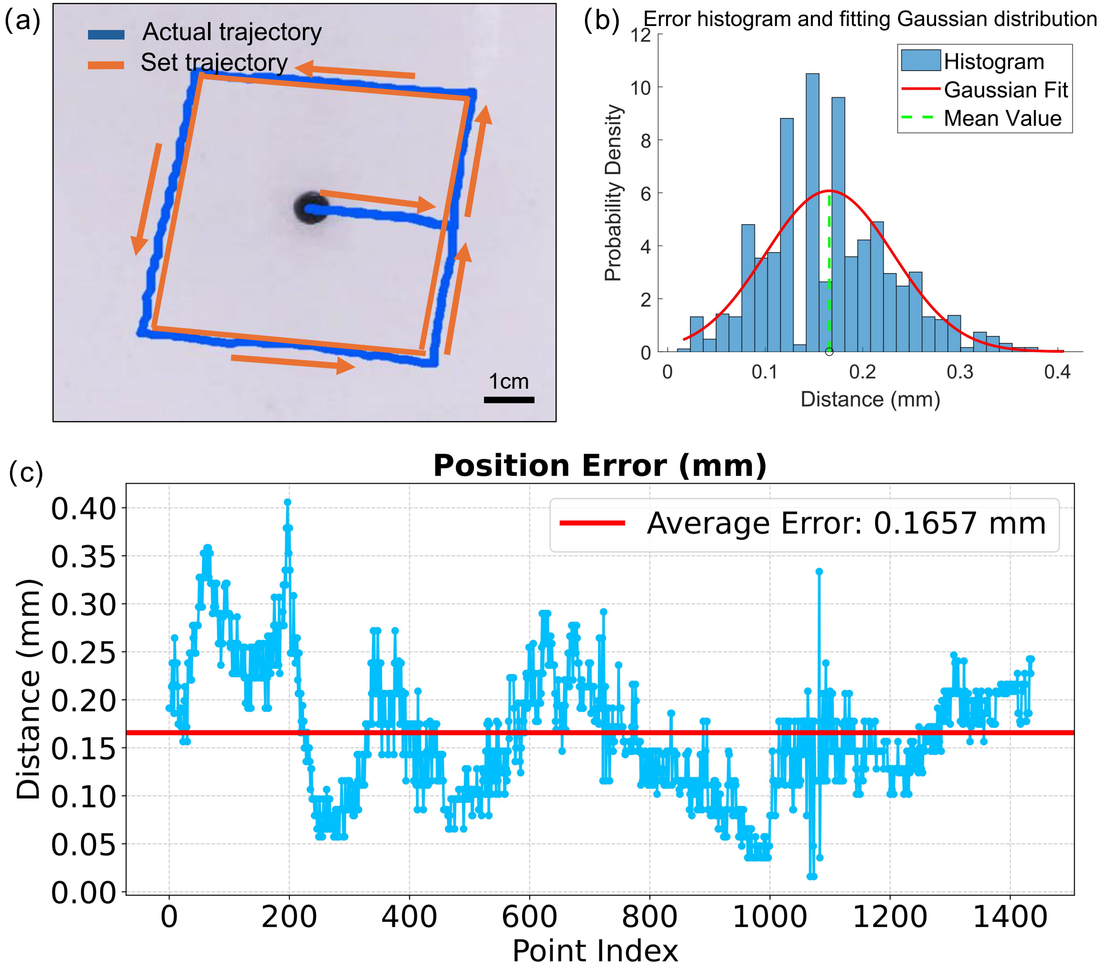

(a)
Actual trajectory
Set trajectory
1cm
(b)
Error histogram and fitting Gaussian distribution
Histogram
Gaussian Fit
Mean Value
Probability Density
Distance (mm)
(c)
Position Error (mm)
Average Error: 0.1657 mm
Distance (mm)
Point Index

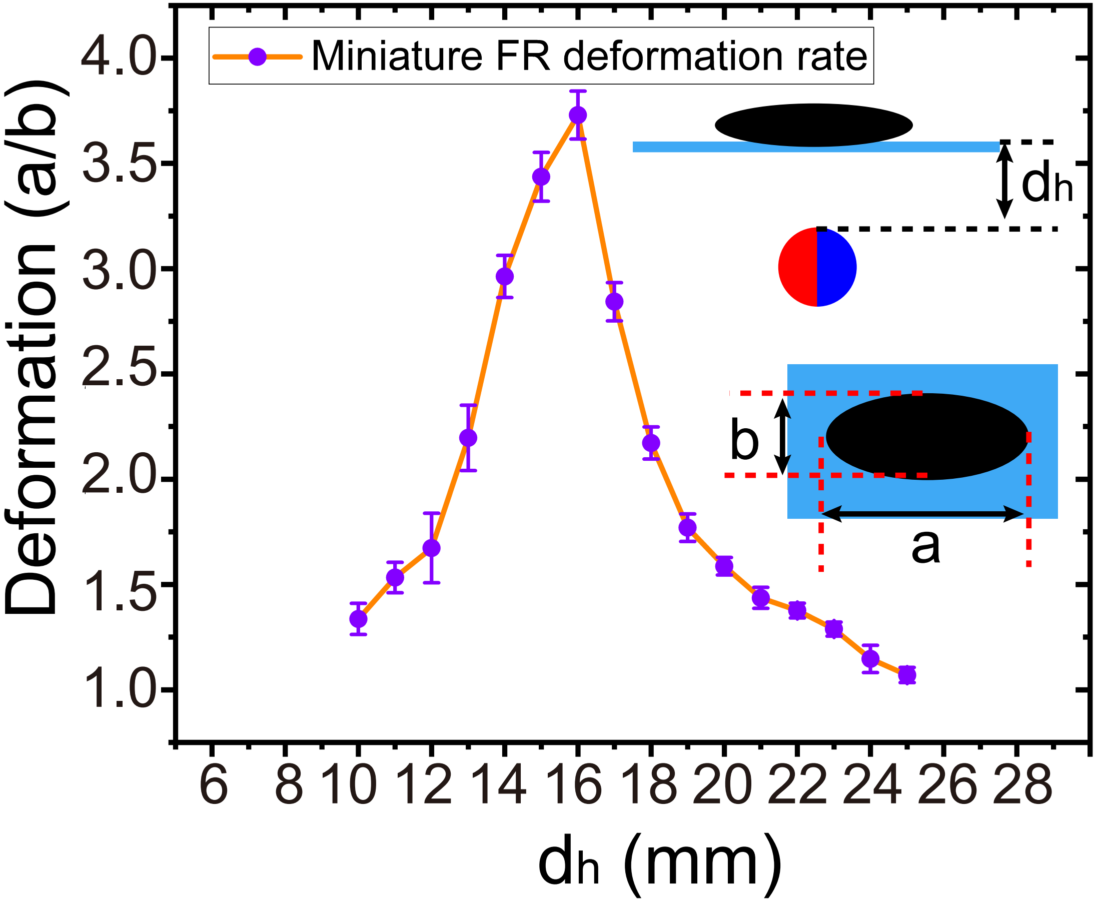

Miniature FR deformation rate
Deformation (a/b)
4.0
3.5
3.0
2.5
2.0
1.5
1.0
6
8
10
12
14
16
18
20
22
24
26
28
dh (mm)
dh
b
a

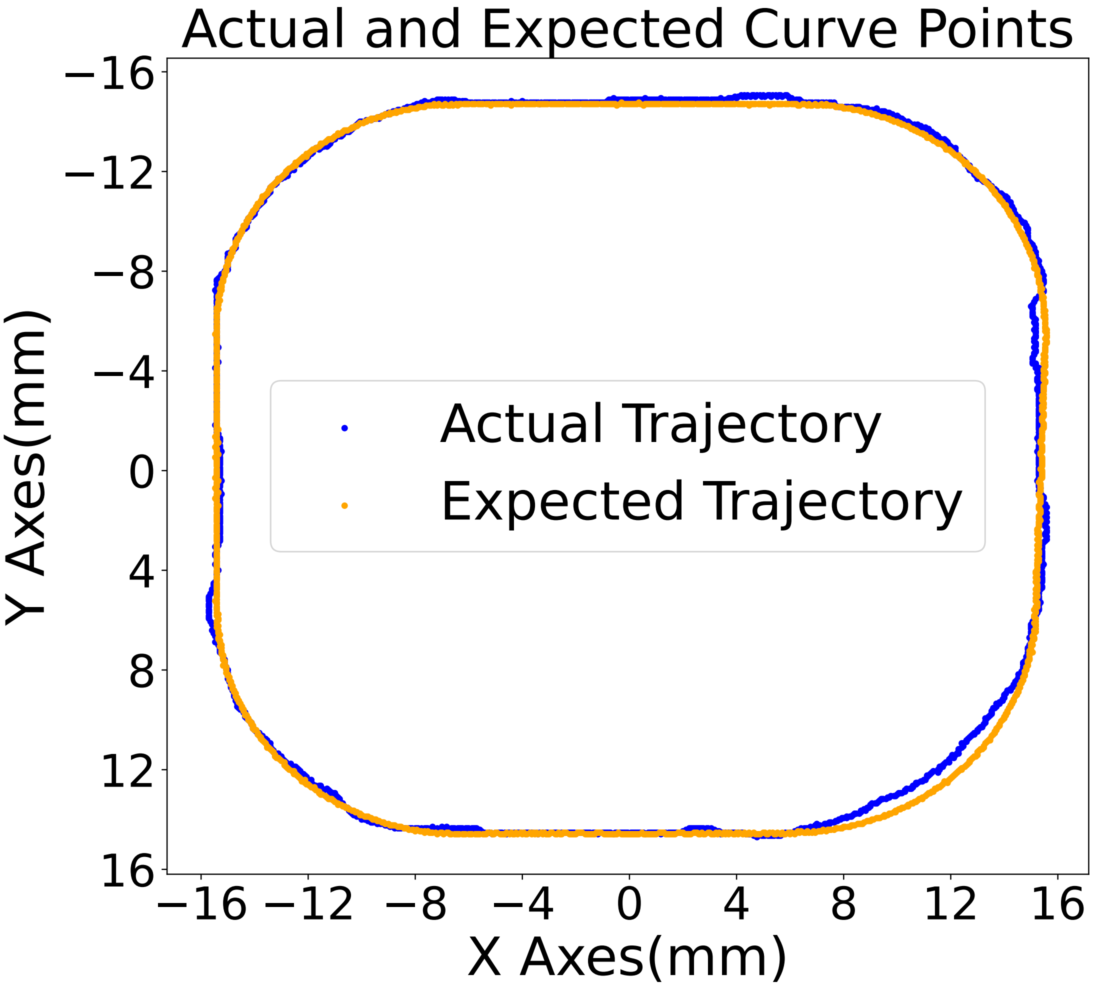
Actual and Expected Curve Points
Actual Trajectory
Expected Trajectory
Y Axes(mm)
X Axes(mm)
−16
−12
−8
−4
0
4
8
12
16

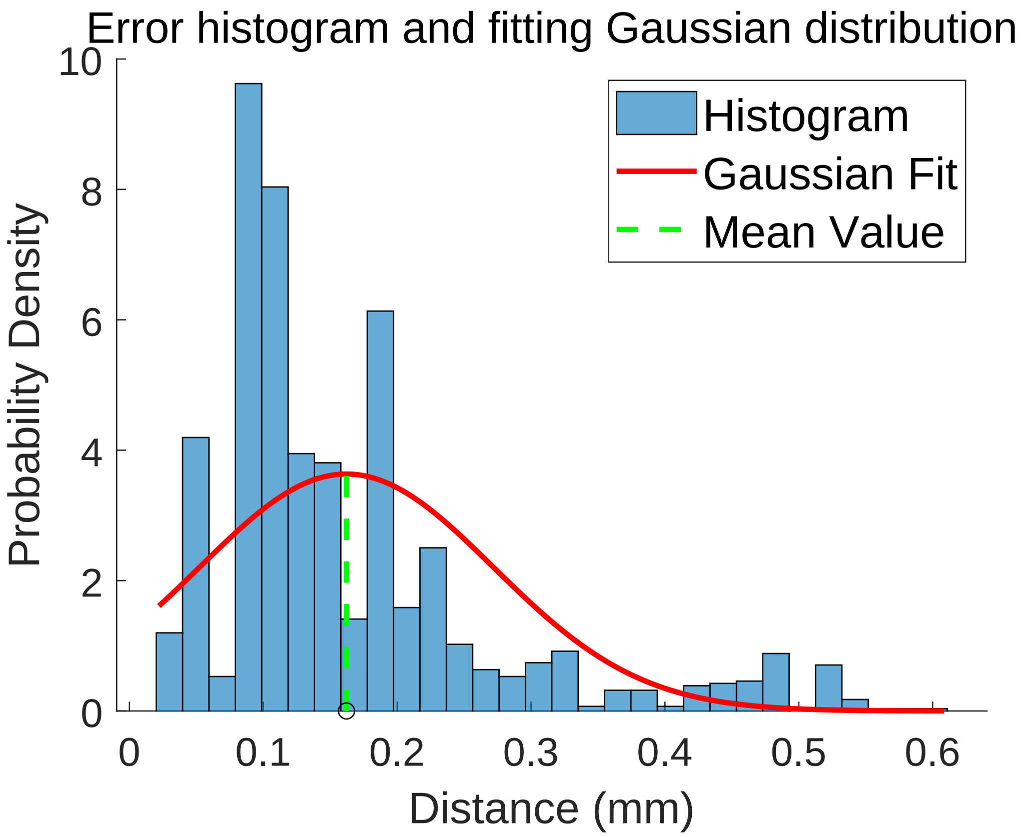
Error histogram and fitting Gaussian distribution
Probability Density
Distance (mm)
Histogram
Gaussian Fit
Mean Value
0
2
4
6
8
10
0
0.1
0.2
0.3
0.4
0.5
0.6

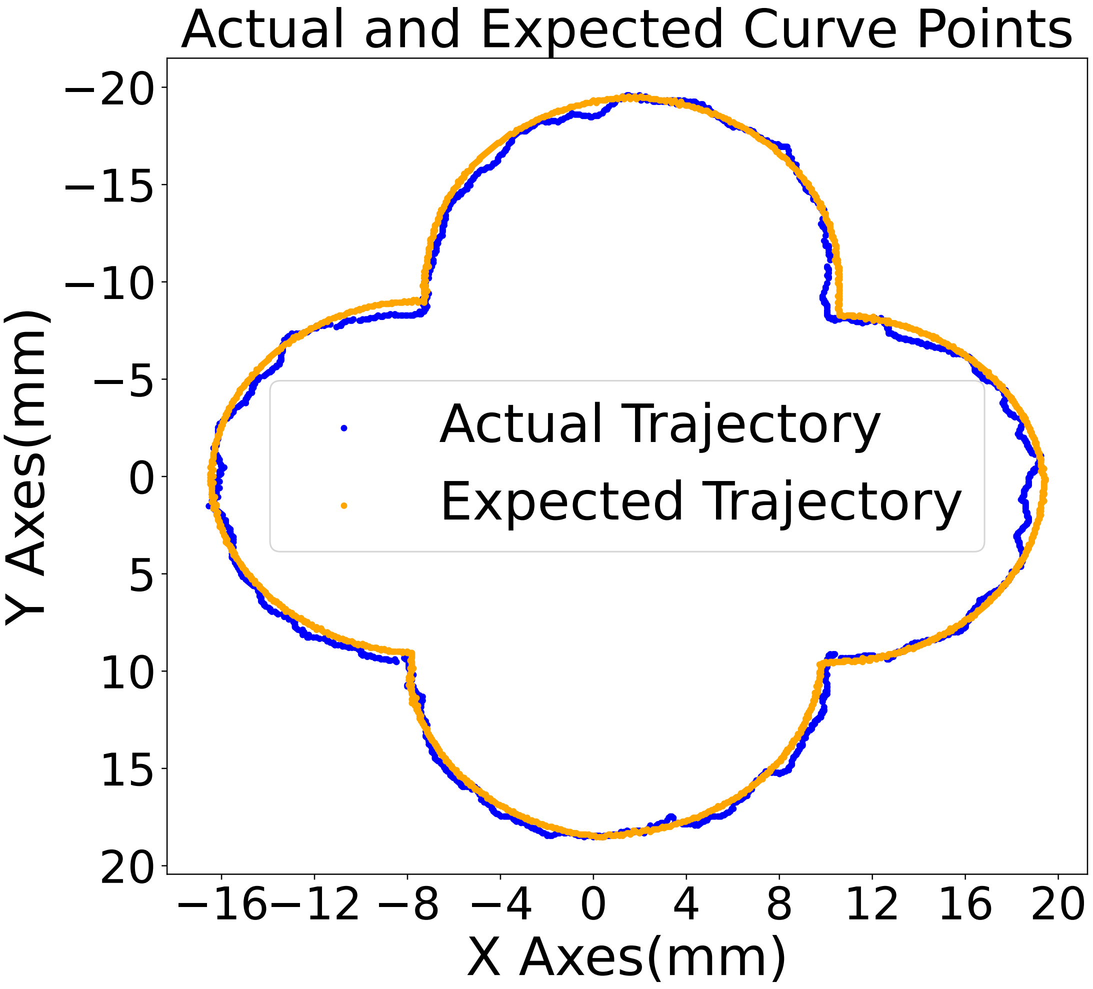

Actual and Expected Curve Points
Actual Trajectory
Expected Trajectory
−20
−15
−10
−5
0
5
10
15
20
Y Axes(mm)
−16
−12
−8
−4
0
4
8
12
16
20
X Axes(mm)

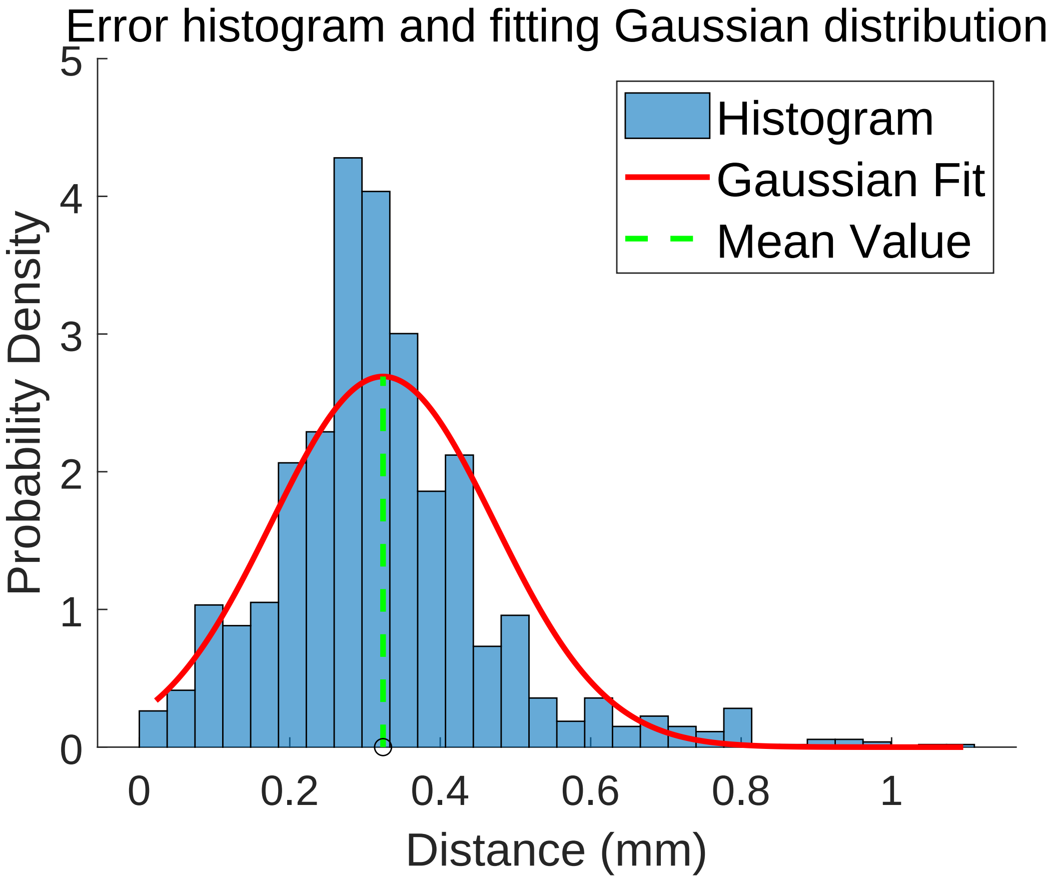
Error histogram and fitting Gaussian distribution
Probability Density
0
1
2
3
4
5
0
0.2
0.4
0.6
0.8
1
Distance (mm)
Histogram
Gaussian Fit
Mean Value

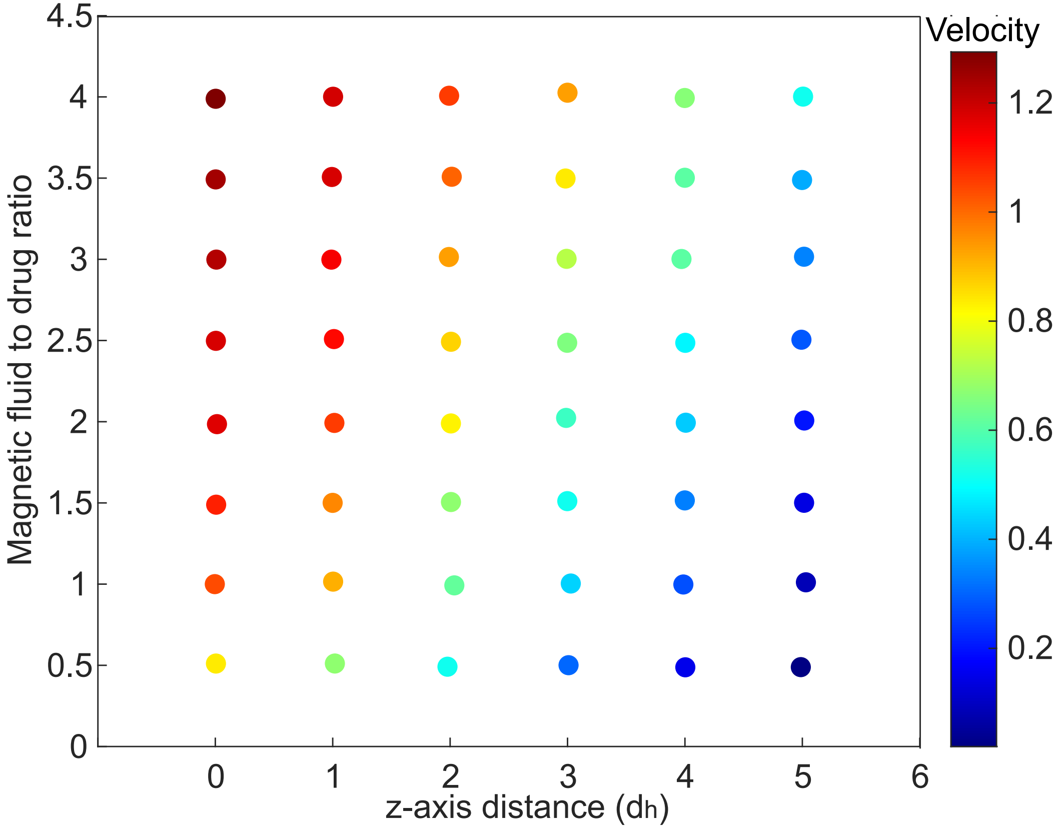

Velocity
1.2
1
0.8
0.6
0.4
0.2
4.5
4
3.5
3
2.5
2
1.5
1
0.5
0
Magnetic fluid to drug ratio
0
1
2
3
4
5
6
z-axis distance (dh)
