# Supplementary material for: Adaptive Ferrofluidic Robotic System with Passive Component Activation Capabilities
Source: Cyborg Bionic Syst. 2025 Jun 24;6:0300. doi: 10.34133/cbsystems.0300 (PMC12187217; doi:10.34133/cbsystems.0300)
Supplement: Supplementary 1 — Figs. S1 to S7 Movies S1 to S11 [file cbsystems.0300.f1.zip › Supplementary Materials.docx]

## Supplementary Materials

Fig. S1. Schematic diagram of the motion of the hybrid magnetic actuation system in the xyz axis

Supplementary Figure 1. (a) The Figure of the hybrid magnetic actuation system achieves displacement along the x-axis. (b)The Figure of the hybrid magnetic actuation system achieves displacement along the y-axis. (c)The Figure of the hybrid magnetic actuation system achieves displacement along the z-axis.

Fig. S2. Schematic diagram of the control system.

Supplementary Figure 2. Schematic diagram of the control system: As shown in the figure, this paper designs a closed-loop control system for the hybrid magnetic control system for locomotion. In the visual imaging module, the Meanshift algorithm and Kalman filter algorithm were synergistically implemented to achieve robust extraction of MFR positions and trajectory smoothing under conditions of external noise interference. In this system, $x_{r}(t),y_{r}(t)$ represents the actual position, $(x_{0},y_{0})$ represents the initial position, and $X(t),Y(t)$ indicates the location of the MFR output by the system, $x_{c}(t),y_{c}(t)$ represent the location where errors exist after being disturbed by environmental noise.

Fig. S3. Parallelogram trajectory of the MFR.

Supplementary Figure 3. In the experiment, the MFR moves within a circular glass petri dish with a diameter of 150 mm. The overall trajectory forms a regular parallelogram with a height and base of 40 mm. The final positioning error is 0.1657 mm, which is relatively small compared to the overall size of the device and the trajectory. A Gaussian distribution graph of these trajectory errors has been plotted and fitted, resulting in an average error of 0.16571mm and a standard deviation of 0.065668. The overall error is relatively small and conforms to a Gaussian distribution.

Fig. S4. The deformation rate of the MFR at different distances.

Supplementary Figure 4. Diagram showing changes in deformation of the MFR relative to the distance ($d_{h}$) between the robot and the top of the SPM. The deformation rate of the MFR based on $d_{h}$ values is defined as the ratio of the relative lengths of the MFR’s major and minor axes (a/b).

Fig. S5. Rounded square trajectory of the MFR.

Supplementary Figure 5. The expected and actual trajectories of the MFR in the rounded square trajectory experiment are visible. A Gaussian distribution graph of these trajectory errors has been plotted and fitted, resulting in an average error of 0.16212mm and a standard deviation of 0.10978. The overall error is relatively small and conforms to a Gaussian distribution.

Fig. S6. Flower-shaped trajectory of the MFR.

Supplementary Figure 6. The figure shows the expected and actual trajectories of the MFR in the flower-shaped trajectory experiment. A Gaussian distribution graph of these trajectory errors has been plotted and fitted, resulting in an average error of 0.32399mm and a standard deviation of 0.14823. The overall error is relatively small and conforms to a Gaussian distribution.

Fig. S7. Scatter plot of the capsule velocity.

Supplementary Figure 7. The scatter plot of the effect of different distances $d_{h}$ between the magnetic control device and the substrate and the varying ratios of the loaded drug and ferrofluid mass, on the capsule’s velocity. Blue represents lower capsule velocity, while red represents higher velocity. As the mass of the ferrofluid in the capsule increases and $d_{h}$ decreases, the response time of the capsule to the magnetic field becomes faster, and the gradient force driving the capsule’s motion becomes stronger, ultimately resulting in a higher capsule speed.

Movie S1. The displacement and workspace of hybrid magnetic actuation devices.

Movie S1.This video demonstrates the motion process of the magnetic actuation system integrated with the triaxial sliding platform. The first part shows the movement of the magnetic actuation system along the x-axis; the second part illustrates its motion along the y-axis; and the third part demonstrates its movement along the z-axis.

Movie S2. Experiment of the MFR following a parallelogram trajectory.

Movie S2.This video demonstrates the locomotion control of MFR in water, driven by the magnetic field gradient of an externally programmed magnetic field.

Movie S3. Experiment of the MFR navigating a complex maze.

Movie S3.This video showcases the orientation control of locomotion for MFR in a complex maze driven by magnetic torque and magnetic gradient forces. Regarding path planning, the A* algorithm is used to achieve the shortest path planning through the labyrinth.

Movie S4. Experiment on controllable deformation passing through narrow channels.

Movie S4.This video demonstrates the size restructuring movement of MFR in a variable-size structure by changing its aspect ratio and reducing the length of its short axis to adapt to passing through narrow gaps.

Movie S5. Experiment on locomotion in the 3D simulated vascular environment.

Movie S5. This video demonstrates the robust locomotion capability of the MFR within a simulated three-dimensional vascular environment. As shown, the MFR achieves stable directional motion along the vascular wall under the control of the hybrid magnetic actuation system. Although partial fluid splitting occurs in high-curvature regions due to magnetic field attenuation from the actuation system and geometric constraints of the vascular structure, the MFR ultimately reconfigures and self-aggregates via the high-gradient magnetic field generated by the permanent magnet. This behavior validates the potential of MFR for biomedical applications, particularly in interventions targeting biological luminal structures.

Movie S6. Experiment of the MFR following a rounded square trajectory.

Movie S6.This video demonstrates the orientation control of locomotion for MFR in water, driven by an externally programmed magnetic field’s magnetic gradient and torque. The MFR moves along a rounded square trajectory, and the MFR’s stretching orientation aligns with the rounded square’s tangent orientation.

Movie S7. Experiment of the MFR following a floral-shaped trajectory.

Movie S7.This video demonstrates the orientation control of locomotion for MFR in water, driven by an externally programmed magnetic field’s magnetic gradient and magnetic rotational field. The MFR moves along a floral trajectory (composed of four semicircles), while the magnetic droplet robot maintains rotational movement around its axis.

Movie S8. Experiment on overcoming simulated stomach folds.

Movie S8.This video demonstrates the joint control of locomotion orientation and deformation of MFR under an externally programmed magnetic gradient field. The MFR maneuvers over the folds and through the crevices of a simulated stomach model, performing tasks such as converging at specific locations, thereby verifying the performance of joint control.

Movie S9. Experiment on gear mechanism engagement and driving.

Movie S9.This video showcases the expansion and validation of the application of MFR as power packs through the design of innovative mechanisms. A magnetic braking gear mechanism has been designed, where the position and orientation control of the MFR is coupled with gear mechanism motion control through magnetic fluid stretching and deformation. This setup drives the engagement and rotation between gear mechanisms as a power pack.

Movie S10. Experiment with power pack delivering items.

Movie S10.This video demonstrates the design of a delivery device that utilizes the MFR as a power pack to deliver items to specific locations. After the complete transport, the robot decouples and separates from the delivery device, verifying its targeted delivery function.

Movie S11. Experiment on targeted capsule delivery and selective blocking.

Movie S11.This video showcases the design of a new type of magnetically actuated ferrofluidic drug capsule. The joint control of the locomotion orientation and deformation of the miniature MFR drive the robot to a specific location. Simultaneously, using the magnetic hyperthermia effect of a high-frequency magnetic field, the dissolution of the ferrofluidic drug capsule is accelerated at the set location, achieving targeted drug delivery. To further simulate real-world conditions and reduce damage to normal tissues, the MFR selectively blocks blood vessels through its deformation, preventing the entry of drugs. This verifies the biomedical effects of the magnetically actuated ferrofluidic drug capsule in human applications, such as chemotherapy embolization.
